# The Importance of New EBMT Criteria on the Diagnosis of Veno-Occlusive Liver Disease in Children

**DOI:** 10.3390/jcm12030826

**Published:** 2023-01-20

**Authors:** Mária Füssiová, Peter Švec, Júlia Horáková, Petr Sedláček, Peter Rohoň, Peter Celec, Ivana Boďová, Jaroslava Adamčáková, Tomáš Sýkora, Veronika Dobšinská, Miroslava Pozdechová, Dominika Dóczyová, Santia Vargová, Alexandra Kolenová

**Affiliations:** 1 Bone Marrow Transplantation Unit, Department of Pediatric Hematology and Oncology, National Institute of Children’s Diseases, Comenius University, 833 40 Bratislava, Slovakia; 2 Department of Pediatric Hematology and Oncology, Charles University Motol, 150 06 Prague, Czech Republic; 3 Department of Hematology and Transfusion, Faculty of Medicine, Comenius University, 812 50 Bratislava, Slovakia; 4 Institute of Molecular Biomedicine, Faculty of Medicine, Comenius University, 811 08 Bratislava, Slovakia

**Keywords:** veno-occlusive disease of the liver, hematopoietic stem cell transplantation, new diagnostic and severity EBMT criteria in childhood, retrospective analysis

## Abstract

Background: Early recognition and specific therapy facilitate a favorable disease course in hepatic venous-occlusive disease (HVOD) following hematopoietic stem cell transplantation (HCT). Diagnostic and classification criteria, published by the European Society for Blood and Marrow Transplantation (EBMT), better account for clinical differences in disease presentation in pediatric populations. Objectives: To compare the course of HVOD in children before and after the implementation of new EBMT criteria. Material and methods: The study retrospectively evaluates 26 HVODs in 179 children treated in a single HCT unit (Slovakia) comparing the period of 2014–2017 using the Baltimore and modified Seattle criteria with the period of 2018–2021, when new EBMT criteria were adopted. Results: No difference in HVOD incidence (11.2% vs. 14.8%, *p* = 0.46) and in time of diagnosis post-HCT (15.6 days vs. 15.7 days, *p* = 0.75) was found. With EBMT criteria we observed more frequent anicteric disease at diagnosis (50% vs. 87.5%, *p* = 0.04), lower serum bilirubin at diagnosis (3.4 mg/dL vs. 1.23 mg/dL, *p* = 0.045), and non-significant trends of shorter defibrotide treatment (21.7 days vs. 15.6 days, *p* = 0.73), decreased mortality (30% vs. 6.2%, *p* = 0.10) and shorter hospitalization (73.1 days vs. 59.6 days, *p* = 0.54). Conclusions: Different time periods around the implementation of new criteria are evaluated, underling that pediatric EBMT criteria for post-transplant HVOD diagnosis appear more sensitive.

## 1. Introduction

Sinusoidal obstruction syndrome (SOS) of the liver, now more commonly called hepatic veno-occlusive disease (HVOD or VOD) is a potentially fatal complication of hematopoietic stem cell transplantation (HCT). The average incidence of HVOD in the pediatric population is in the range of 5–20% (5.3–13.7% in adults) [1,2,3,4,5,6,7,8,9,10]. The incidence of the anicteric form of HVOD at the time of diagnosis in children is 29–32% and in adults, around 12% [2,11]. In 20–30% of affected pediatric patients, the disease progresses to multi-organ dysfunction or failure (MOD/MOF) with a high mortality rate (>80–85%) [2,3,11,12,13,14,15].

Risk factors of post-transplant HVOD are generally classified as either patient- and disease-related or transplantation related (e.g., active viral hepatitis or total body irradiation-based myeloablative conditioning). There are a number of pediatric conditions, in particular, younger age (liver immaturity) and underlying pediatric disorders such as hemophagocytic lymphohistiocytosis, X-linked adrenoleukodystrophy, Griscelli syndrome, X-linked lymphoproliferative disease, neuroblastoma, osteopetrosis, or thalassemia (preexisting hepatomegaly and hepatopathy, iron overload and/or liver fibrosis), which may enhance the likelihood of HVOD [2,4,15,16,17].

Due to the different characteristics of HVOD in pediatric and adult populations, in 2017, the European Society for Blood and Marrow Transplantation (EBMT) issued new recommendations for the diagnosis of HVOD in children. Based on new knowledge, an international panel of experts revised and expanded those recommendations, issuing a new consensus recommendation for the classification of HVOD in children, adolescents, and young adults in 2019 (Table 1). EBMT defines the pediatric population as persons under 15 years of age, and adolescents and young adults as persons aged 15–25 years [11]. The new pediatric criteria for the diagnosis of hepatic SOS/VOD have a grade of recommendation 2A [17,18]. They do not have a time limit for establishing the diagnosis: they confirm the diagnosis of HVOD, but they do not differentiate between the early and late forms of the disease (unlike the old criteria such as the Seattle or Baltimore criteria—Table 2).

HVOD is a clinical diagnosis based on physical assessments. Historically used Seattle and modified Baltimore criteria were not designated for the pediatric population [2,3,15,17,19,20]. These criteria failed to account for anicteric HVOD, which may occur more frequently among pediatric patients [21]. In addition to obligatory hyperbilirubinemia, children’s other shortcomings of these criteria, such as the time limit of 20 and 21 days, respectively, and the arbitrary weight gain of 2 and 5%, are present [22]. Moreover, the criteria to not meet the special differences of individual periods of childhood need to be mentioned (e.g., children are more prone to rapid weight changes, partially due to iatrogenic fluid overload) [8,11].

The pediatric EBMT diagnostic criteria aimed predominantly at clinical aspects that have not been considered so far, emphasize the speed of disease progression, and hemodynamic instability as confirmed by ultrasonography (a more objective assessment of hepatomegaly or ascites) [10]. Primarily hyperbilirubinemia is not an obligatory criterion for pediatric HVOD (high percentage of anicteric disease in children). Numerous reports demonstrated that hyperbilirubinemia >2 mg/dL is rather a criterion for poor outcomes so the conservative criteria with mandatory hyperbilirubinemia seem to cause an unnecessary delay in HVOD diagnosis [8]. The pediatric EBMT criteria account for anicteric HVOD and use a baseline bilirubin measurement from which to assess diagnosis [21]. In addition, more important than the absolute value are the dynamic changes in bilirubin level. Hyperbilirubinemia, as well as hepatomegaly and ascites, are frequent pre-transplantation conditions. For the HVOD diagnosis precise pre-HCT baseline measures of the liver and ascites are mandatory [22]. Post-transfusion refractory thrombocytopenia (RT) is now increasingly recognized as a sensitive predictor of HVOD and was therefore integrated into the new EBMT criteria [11,23]. Taken together, the revised diagnostic criteria justify the differences in various aspects of the disease between children and adults and offer the potential for early diagnosis [8].

The severity of HVOD is evaluated according to clinical symptoms and laboratory testing during and after the resolution of the disease. For the last three decades, McDonald criteria were used to classify the severity of HVOD (unanimously for children and adults, Table 3) [24].

The new severity stratification guideline takes into account the above diagnostic criteria, the progression of hepatic, renal, and respiratory failure, and the degree of encephalopathy (Table 4). If a patient fulfills 2 or more criteria in 2 different categories, they must be classified in the most severe category. If the patient meets all the criteria within one grade, they are also assigned to a higher grade of disease severity. The kinetics of the evolution of cumulative symptoms within 48 h predicts a severe form of the disease. Hyperbilirubinemia in children is frequently either absent or found only in the advanced stage of HVOD [17]. Bilirubin greater than or equal to 2 mg/dL defines severe HVOD in children and predicts an increased risk of MOD and death. Doubling of bilirubin from an individual baseline within 48 h should be considered a sign of severe HVOD, too. Rising transaminases after the diagnosis of HVOD reflect hepatocyte failure and advanced-stage disease (grade 4). Elevation of γ-glutamyl transferase may reflect hepatocellular damage. The need for replacement of coagulation factors after the established diagnosis of hepatic VOD, with no response to vitamin K administration, indicates consumptive coagulopathy associated with hepatic failure. Persistent post-transfusion RT for >7 days, despite early therapeutic intervention, indicates severe disease. The need for paracentesis to release abdominal pressure and alleviate respiratory distress is a criterion of HVOD grade 4. Severe HVOD, resulting in MOD or MOF, is characterized by pulmonary and/or renal dysfunction and/or encephalopathy. Any combination of organ failure in HVOD classifies a very severe disease and predicts an increased risk of death and the need for prolonged treatment [11,17,19].

The implementation of new diagnostic and classification EBMT criteria into practice offers the possibility of early therapeutic intervention, which has an impact on the course, severity, and prognosis of HVOD. According to up-to-date studies, pre-emptive intervention in post-transplant HVOD is crucial for a patient’s survival [8]. The treatment is complex. In addition to preventive measures, it includes supportive symptomatic therapy and causal treatment with defibrotide. Defibrotide is currently the sole drug approved in the United States and the European Union for treating subsets of severe HVOD after HCT in patients older than one month [3,4,7,25,26,27,28].

On 13 June 2022, the European Medicines Agency (EMA) and the State Institute for the Control of Medicinal Products in Slovakia issued a recommendation based on which defibrotide should not be used as a prophylaxis for venous-occlusive liver disease. Study 15-007 (198 pediatric and 174 adult patients) comparing defibrotide in combination with best supportive care (BSC) versus BSC alone for prophylaxis of HVOD after HCT was stopped due to lack of efficacy. There was no effect on the primary efficacy endpoint—VOD-free survival up to day 30 after HCT [29].

In the presented study, we aimed to explore the course of HVOD in children before and after the implementation of new EBMT criteria in our center. We focused on describing clinical and laboratory features of HVODs together with outcome parameters in both analyzed periods.

## 2. Material and Methods

Our study retrospectively analyzes HVOD in children treated in the Bone Marrow Transplantation Unit (BMTU)—Department of Pediatric Hematology and Oncology Comenius University at the National Institute of Children’s Diseases in Bratislava in the period of 2014–2021. Patients that are 19 years of age or younger were divided into two groups: those treated between 2014 and 2017, when criteria from Baltimore and Seattle were used to diagnose HVOD, and those treated between 2018 and 2021 when new pediatric EBMT recommendations for diagnosis and classification of HVOD had been adopted. Patients with HVOD were treated with defibrotide at a dose of 25 mg/kg/day, administered intravenously, every 6 h, in a 2-h infusion.

The aim of the study was to compare the characteristics of the disease in individual periods: the difference in incidence, time of diagnosis post-HCT, severity of the disease, length of treatment, survival, mortality, and length of hospitalization in patients with a confirmed diagnosis of HVOD.

Time to diagnosis was defined as the time from the date of the transplantation to the date of diagnosis of HVOD for each patient based on diagnostic criteria. The interval between the diagnosis and the start of defibrotide treatment was less than 24 h. The duration of defibrotide treatment (from the day of diagnosis until complete remission) was determined by individual presentation of “dynamic” and “static” HVOD criteria [18]. Overall survival was defined as the time from the date of transplant to the date of death or the date of last follow-up (day +100 from HCT).

### 2.1. Statistical Methods

Statistical analysis was performed using XLStatistics 5.76 performing the Chi-square and Mann-Whitney tests. *p*-values less than 0.05 were considered significant. Overall survival was estimated by the Kaplan-Meier method.

### 2.2. Characteristics of Study Cohort

A total of 179 consecutive pediatric patients were included in the study, in which 197 HCT procedures (135 allogeneic and 62 autologous transplantation) were performed. The clinical and transplantation characteristics of the enrolled patients are summarized in Table 5. All patients, for whom defibrotide treatment was started, but later HVOD was not confirmed, were excluded from the study. Patients treated with prophylactic defibrotide in the peritransplantation period for high risk of HVOD, were also excluded. Detailed analysis was focused on 26 patients diagnosed with HVOD post-HCT during the above-mentioned period (we did not analyze in more detail patients with suspected but later unconfirmed HVOD).

In the period from 2014 to 2017, modified Seattle criteria and/or Baltimore criteria were used to diagnose HVOD, and McDonald criteria were used to classify the severity of the disease. This period includes 82 patients who underwent 89 transplantations (7 patients had allogeneic retransplantation). The indications for transplantation in the HVOD cohort were B-acute lymphoblastic leukemia, myelodysplastic syndrome, juvenile myelomonocytic leukemia, neuroblastoma, Wiskott-Aldrich syndrome, congenital amegakaryocytic thrombocytopenia and very severe aplastic anemia. Nine (90%) of 10 patients with HVOD received allogeneic HCT. Among these patients, the stem cells were derived from bone marrow in 3 cases and from peripheral blood in 5 cases. One patient had a combined source of hematopoietic stem cells (bone marrow and umbilical cord blood from the HLA-identical sister). Conditioning regimens, as a well-known risk factor for hepatic VOD, were also assessed. All patients, except for three, that were diagnosed with HVOD, received a myeloablative regimen prior to HCT.

In the period of 2018–2021, the new EBMT recommendations from 2017 to 2019 were used for the diagnosis and classification of HVOD. During this period, 97 patients who underwent 108 transplantations (3 patients had allogeneic retransplantation and 4 patients had repeated autologous HCT) were included. The indications for transplantation in the HVOD cohort were most commonly solid tumors (56.2%), mainly neuroblastoma (50%). Among the HVOD patients, in contrast to the period of 2014–2017, autologous transplantations dominated (56.2%). Other patients with veno-occlusive disease (53.8%) had allogeneic HCT from an unrelated donor. Twelve of them (75%) received peripheral blood stem cells, the other patients had bone marrow. Similar to the previous period, in these patients, the myeloablative conditioning regimen dominated (87.5%).

## 3. Results

**Figure 1:**
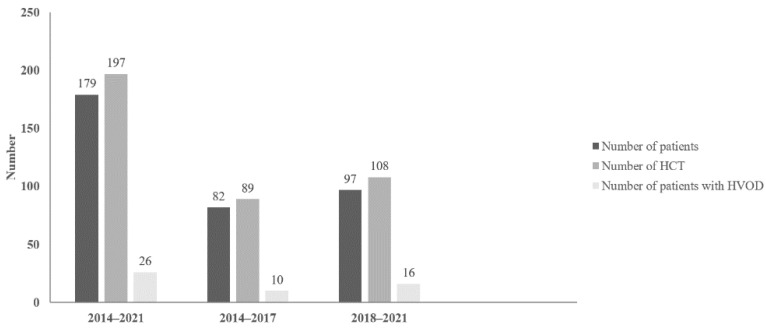
Incidence of hepatic veno-occlusive disease (HVOD). HCT = hematopoietic stem cell transplantation, HVOD = hepatic veno-occlusive disease.

Our retrospective analysis confirmed the diagnosis of HVOD in 26 patients (13.1% of transplantations and 14.5% of patients) (Figure 1).

From 2014 to 2017, 10 patients (11.2% of transplantations), with a median age of 10.3 years, were diagnosed with veno-occlusive liver disease according to the Baltimore criteria and/or the modified Seattle criteria (Table 6). Seven patients (70%) had a mild form of the disease (grade I and II). A severe HVOD was detected in 3 patients (30%). The median time to diagnosis was 15.6 days from HCT. In this period, early HVOD predominantly occurred (90%). Only 1 patient had a late-onset form of the disease (diagnosis was established 35 days after HCT). At the time of diagnosis, five of 10 patients (50%) had an anicteric disease. The serum bilirubin concentration at the time of diagnosis was in the range of 0.42–13.72 mg/dL (median: 3.4 mg/dL). Despite the significant proportion of the anicteric HVOD at diagnosis, most patients developed hyperbilirubinemia. The maximum serum bilirubin concentration was on average 9.58 mg/dL (0.42–31.82 mg/dL). The earliest and most consistent symptom of HVOD in this cohort was post-transfusion RT. It was observed in 90% of children, commonly as the first sign of HVOD. At the time of diagnosis, 6 patients (60%) met the criteria for hyperbilirubinemia. At diagnosis, 80% of patients had weight gain, all patients had hepatomegaly above baseline, and 80% of patients had ascites verified by ultrasonography (US). Doppler US showed signs of HVOD in 50% of children. Despite treatment with defibrotide and comprehensive, intensive supportive care, some patients developed signs of MOD/MOF. Fifty percent of children with HVOD had coagulopathy 50% of children required oxygen inhalation treatment, 30% of patients had to be on ventilatory support, 30% of children had encephalopathy and 20% of children had renal insufficiency. Complete resolution of the liver damage with defibrotide treatment was noted in 70% of patients. The average duration of defibrotide treatment was 21.7 days (range 5–43 days). Overall survival on day +100 after HCT in this period was 70%. In the analyzed cohort, three (30%) patients died (day +15, +70, +77). The immediate cause of death for all of them was microbiologically proven mycotic systemic infection. The length of hospitalization in the period ranged from 30 to 134 days, with an average of 73.1 days.

In the period of 2018–2021, 16 patients (14.8%), median age: 5 years, were diagnosed with veno-occlusive liver disease according to new EBMT criteria. According to the new severity classification, in this period 9 patients (56.2%) had a mild form of the disease (grade I and II), and less than half of the HVOD cases met the criteria of severe and very severe HVOD (7/16, 43.7%). The median time post-HCT at the moment of HVOD diagnosis was 15.7 days (2–23 days). The early form of HVOD dominated in this period as well (81.2%). At the time of diagnosis, 14 children (87.5%) had an anicteric disease. Serum bilirubin level at the diagnosis was in the range of 0.17–5.11 mg/dL. The average concentration of bilirubin compared to the previous period was significantly lower: 1.23 mg/dL. The peak serum bilirubin level in the HVOD cohort varied from 1.04–30.41 mg/dL (median 4.29 mg/dL). Only nine patients (56.2%) in this period met the criteria for the elevation of serum bilirubin at the time of diagnosis. The initial clinical VOD symptoms were remarkable. Refractoriness to platelet transfusions was present in 13 patients (81.2%). Seven children (43.7%) had a weight gain. Ultrasound-confirmed ascites were present in 14 patients (87.5%), and 12 patients (75%) were diagnosed with hepatomegaly above baseline. Doppler US at the time of diagnosis showed signs of HVOD in 13 children (81.2%), 31.2% higher than in the previous period. Despite defibrotide treatment and supportive care, some patients again developed signs of MOD and/or MOF. Twenty-five percent of children had coagulopathy, 12.5% of children required oxygen inhalation treatment and had encephalopathy, one patient required mechanical ventilation for respiratory insufficiency, hemodialysis for renal insufficiency, and molecular adsorbents recirculating system elimination method for the hepatic failure. Complete resolution of the liver damage on defibrotide treatment was recorded in 15 children (93.7%). The average duration of defibrotide treatment was 15.6 days (range 7–30 days). Overall survival on day +100 after HCT in the period was 93.7%. One patient (6.2%) died on day +39 after HCT due to a very severe HVOD with multiorgan failure and possible development of pulmonary VOD. The length of hospitalization in this period ranged from 39 to 88 days, the average is 59.6 days.

Results of the retrospective analysis and statistical evaluation of the determined parameters are presented in Table 6. In the period of 2018–2021, the incidence of HVOD was similar (11.2% vs. 14.8% of transplantations, with a non-significant 1.3-fold increase, *p* = 0.46). The time of HVOD diagnosis from HCT between the groups was without statistical significance (15.6 days vs. 15.7 days, *p* = 0.75), this was identical to defibrotide treatment initiation in all patients. The severity of HVOD between the groups could not be compared, as the criteria for classifying the severity of the disease were different.

**Figure 2:**
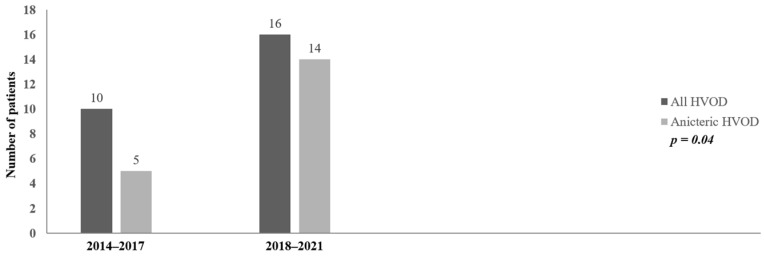
Incidence of anicteric HVOD at diagnosis. HVOD = hepatic veno-occlusive disease.

The occurrence of an anicteric form of HVOD at the time of diagnosis was high in both periods and was significantly higher in the period of 2018–2021 (50% vs. 87.5%, *p* = 0.04) (Figure 2). This is an important and remarkable finding of the study.

**Figure 3:**
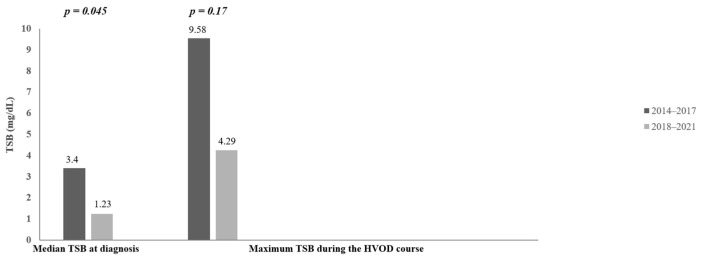
Median of total serum bilirubin at diagnosis and median of maximum serum bilirubin level during the HVOD course. TSB = total serum bilirubin, HVOD = hepatic veno-occlusive disease.

The average serum concentration of unconjugated bilirubin at the time of diagnosis was lower in the period of 2018–2021 (3.4 mg/dL vs. 1.23 mg/dL, *p* = 0.045) (Figure 3). This is a significant result of our research. The average maximum serum concentration of unconjugated bilirubin appeared also lower in the period of 2018–2021 (9.58 mg/dL vs. 4.29 mg/dL). It is a 2.2-fold decrease compared to the period of 2014–2017, but the difference is not statistically significant (*p* = 0.17) (Figure 3).

**Figure 4:**
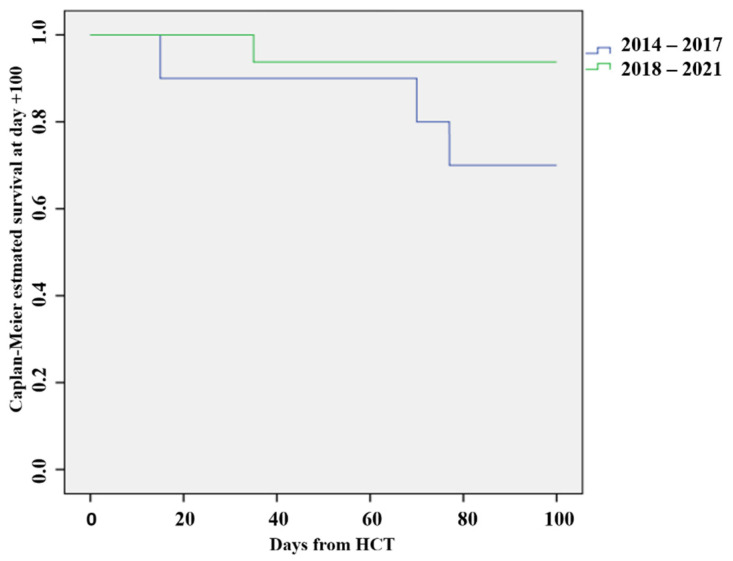
Overall survival at day +100 after HCT. HCT = hematopoietic stem cell transplantation.

There was a trend to the shorter median duration of defibrotide treatment in the period of 2018–2021 (21.7 days vs. 15.6 days, *p* = 0.73). Similarly, there were trends of better overall survival on day +100 in the period of 2018–2021 (70% vs. 93.7%), in contrast to the trends of increased mortality on day +100 in the period from 2014 to 2017. The differences are not statistically significant (*p* = 0.10) (Figure 4).

The average length of hospitalization appeared shorter in the period of 2018–2021 (73.1 days vs. 59.6 days)—a reduction of 13.5 days (1.2-fold decrease) seems remarkable; however, the difference is not statistically significant in the small cohort (*p* = 0.54).

## 4. Discussion

Our retrospective single-center study evaluates the important periods of HVOD diagnosis in pediatric hematopoietic stem cell transplantation during the transition to the new EBMT diagnostic and classification criteria for HVOD in children, adolescents, and young adults.

The incidence of HVOD differs by the criteria used for diagnosis. Consistent with the published literature, we observed a trend of a slightly increased incidence of HVOD when new diagnostic criteria were used [19] (not statistically significant in our cohort). According to different authors, the difference in incidence was found higher. Modified Seattle criteria compared to the Baltimore criteria increase the incidence of HVOD fourfold (10.8% vs. 2.5%), and EBMT criteria compared to the modified Seattle criteria about twice as much (unpublished data: professor Corbacioglu at the EBMT conference, 2020). Similarly, according to the prospective analysis of Szmit et al., the incidence of HVOD diagnosed using EBMT criteria in children (aged from 2 months up 21 years) increased 1.8 times compared to the modified Seattle criteria (8.9% vs. 4.9%, *p* = 0.021) [8]. Ragoonanan evaluated the incidence of HVOD using the historic Baltimore criteria, the modified Seattle criteria, and the new pediatric EBMT criteria in a cohort of 226 patients (aged from 1 year up 28 years). The highest incidence of HVOD was with the use of EBMT criteria (6.6% vs. 12.3% vs. 15.9%) [21]. In 2014 and 2018, the incidence of hepatic VOD was 1.94 times more (9.52% vs. 18.47%) in pediatric patients, according to the nationwide data of the Korean Health Insurance Review and Assessment Service [10].

The increase in HVOD incidence using the modified Seattle and EBMT criteria compared to the Baltimore criteria can be explained by the fact that the Seattle and EBMT criteria, unlike the Baltimore criteria, do not consider hyperbilirubinemia as the main criterion [18]. In addition, most authors compare cohorts with simultaneous application of different HVOD diagnostic criteria, our study evaluates these criteria in real time. The increase in incidence may be associated with possible “overdiagnosis” of HVOD. Therefore, great emphasis must be placed on differential diagnosis (excluding bacterial, fungal, viral infections, and other conditions) [2,26,29,30,31,32,33,34,35]. Our relatively constant incidence in the BMTU Bratislava does not support the concern of overdiagnosing HVOD due to the EBMT criteria.

In our study, an increased incidence was noted in patients with neuroblastoma, which is a disease with a high occurrence of HVOD after busulfan conditioning. Female sex, young age, malignancy, and myeloablative conditioning regimen still represent significant risks of HVOD according to the analysis (in accordance with the data in specialized literature) [4,5,11,15,16,27,32,36,37,38,39].

There was no difference in the time of diagnosis after HCT between the two periods. However, in a retrospective study by Ragoonanan et al., the median time to diagnosis varied with the diagnostic criteria used. In the analysis of 226 pediatric patients, the pediatric EBMT criteria were associated with the earliest time to diagnosis of HVOD at a median of 10.5 (2–22) days post-HCT versus 15 days with both the modified Seattle (6–58 days) and Baltimore criteria (7–19 days) [21]. Szmit et al. published a similar result. The median delay in diagnosis of HVOD using the modified Seattle criteria compared with the EBMT criteria was 3 days (0–11 days) [8].

The comparative prospective analysis of Szmit et al., similar to our study, confirmed a significantly higher incidence of anicteric disease in children (only 20% of patients had hyperbilirubinemia at the time of diagnosis, the median serum bilirubin at the time of diagnosis established according to the modified Seattle criteria was 2.83 mg/dL, and according to the EBMT criteria 1.2 mg/dL, *p* = 0.0001) [8]. The proportion of anicteric HVOD in the study from Ragoonan was the same (61%) [21].

Differences in diagnostic criteria may result in a potential delay in time to diagnose and initiate the defibrotide treatment [21]. Regular clinical examination and monitoring of weight and fluid balance remain the basic standard for early diagnosis and monitoring of HVOD. Thrombocytopenia with rapid consumption of transfused platelets, as a valid and early HVOD diagnostic criterion, is frequently observed in children (90% and 81.2% of patients in our study). HVOD should be suspected in any patient with RT after the exclusion of other causes of consumptive thrombocytopenia, especially if they require >7 mL/kg/day of platelets [11,23,40,41,42].

The most practical imaging tool to confirm hepatomegaly and ascites is ultrasound (in our study classical US showed signs of HVOD in 100% and 93.7% of children respectively in the two studied periods and Doppler US signs were presented in 50% and 81.2% of patients at diagnosis), which allows regular detection of liver size and/or the presence of fluid in the abdominal or chest cavity. The sonographic criteria of HVOD using classic and Doppler ultrasonography (Lassau criteria, Hok-US10, US-17), as well as liver stiffness measured by elastography, are coming to the fore in establishing the diagnosis (our study shows dominant ultrasound signs of HVOD at diagnosis) [2,7,35,43,44,45,46]. In 2019, Ravaioli et al. published a multidisciplinary diagnostic procedure for the diagnosis of HVOD [26]. A similar procedure should be developed in each pediatric bone marrow transplantation center as part of standard procedures.

The overall survival and mortality between the periods did not differ significant in our study. The trend toward higher mortality in the period of 2014–2017 may be due to a higher average serum bilirubin concentration at the time of diagnosis and a higher maximum serum bilirubin concentration during the course of the disease (hyperbilirubinemia is a poor prognostic factor of HVOD [8]). The trend of 4.8-fold decrease in mortality per day +100 in the period of 2018–2021 may also be related to the lower median serum bilirubin concentration at diagnosis (statistically significant) and the lower average maximum serum bilirubin concentration in this period. Further, in the latter period, we assume better supportive care (e.g., using a prophylactic strategy of oral ursodiol or better ultrasound scanning skills) leads to improvement of the HVOD management (as indicated by the lower serum bilirubin concentration at diagnosis, lower peak serum bilirubin, trends of decrease in the length of defibrotide treatment, better survival and lower mortality on D+100, shorter hospitalization).

Similar results were published by Szmit et al. in a study comparing EBMT and modified Seattle criteria. EBMT criteria reduce mortality and length of hospitalization (overall survival in case of EBMT criteria was 88%, in case of modified Seattle criteria 56.2%, *p* = 0.008; length of hospitalization in case of EBMT criteria was 42 days, and in case of modified Seattle criteria 54 days, *p* = 0.009) [8]. Worse survival in pediatric patients with HVOD using the Baltimore criteria or the modified Seattle criteria suggests that waiting for the presence of hyperbilirubinemia delays diagnosis and defibrotide treatment and thus may lead to disease progression and development of MOD/MOF [1,13,14].

Our study has some limitations, such as a retrospective, single-center design, a relatively small sample size, an incomplete medical record, and heterogeneity of the compared groups in terms of age and primary diagnoses. Differences in supportive care and availability of defibrotide in two periods can impact the outcome parameters, too.

In Slovakia, there has been no nationwide report of the incidence of pediatric HVOD (our center is the only pediatric bone marrow transplantation unit), and no large transplantation cohort studies are available regarding pediatric HVOD or other post-HCT complications. Our single-center retrospective study revealed two statistically significant findings: anicteric HVOD was more frequent and serum bilirubin was lower at diagnosis. The other parameters were numerically but not statistically different. At the same time, these results also make it possible to set goals for the further improvement of HVOD management including strict adherence to international recommendations in the diagnosis and classification of HVOD, analysis of risk factors of the disease before HCT, using the Center for International Blood and Marrow Transplant Research (CIBMTR) [47] and Endothelial Activation and Stress Index (EASIX) [48] score, monitoring of HVOD biomarkers [3,4,13,31,49,50,51], the introduction of ultrasound diagnostic score and elastography [2,7,35,43,44,45,46] into practice, regular education of staff about HVOD, regular evaluation of results.

## 5. Conclusions

Veno-occlusive disease of the liver is a serious, potentially life-threatening disease. The incidence of HVOD in pediatric patients after HCT has not decreased over the past decade, despite significant advances in transplant-related care. New international recommendations for the diagnosis and classification of the severity of HVOD in children, adolescents, and young adults take into account the specific characteristics of childhood. Our study suggests that these recommendations may better promote early diagnosis and early treatment of the disease, compared to older guidelines, impacting the severity of the condition and the prognosis of patients. Further studies with larger patient cohorts are necessary to confirm that these recommendations indeed significantly improve the outcomes parameters of patients suffering from HVOD post-HCT.

## Figures and Tables

**Table 1:** New diagnostic criteria according to European Society for Blood and Marrow Transplantation (EBMT) for hepatic veno-occlusive disease (HVOD) in children.

Presence of ≥2 of the Following Criteria (Excluding Other Potential Diagnoses):
Thrombocytopenia refractory to transfusion (need for ≥1 transfusion of platelets per day)—corrected count increment of platelets (CCI) less than 5000–7500/µL one hour after transfusion of fresh platelets (not older than 72 h), of the same type (it is not appropriate to compare aphaeretic preparation with pooled one), compatible in the AB0 system, the examination must be performed repeatedly (at least two or more times). Otherwise unexplained weight gain for three days following despite diuretic therapy or weight gain of 5% above baseline. Hepatomegaly—absolute enlargement of the liver by 1 cm in the midclavicular line; if baseline liver size (the size of the liver before HCT) is not available, hepatomegaly will define as the liver enlargement of 2 standard deviations for age (on US, CT or MRI). Ascites—confirmed in US, CT, and MRI examination. Increase of serum bilirubin values from baseline for three consecutive days or serum bilirubin concentration ≥2 mg/dL within 72 h.

CT = computed tomography, MRI = magnetic resonance imaging, US = ultrasound, HCT = hematopoietic stem cell transplantation.

**Table 2:** Comparison of Seattle and Baltimore HVOD diagnostic criteria.

Criteria	Original Seattle Criteria	Modified Seattle Criteria	Baltimore Criteria
Time after HCT	Before D+30	Before D+20	Before D+21
Number of symptoms for diagnosis	≥2	≥2	Hyperbilirubinemia and ≥1 of the following symptoms
Symptoms	Hyperbilirubinemia	Hyperbilirubinemia >34 µmol/L (>2 mg/dL) and ≥1 another symptom	Hyperbilirubinemia >34 µmol/L (>2 mg/dL)
	Hepatomegaly and right upper quadrant pain of liver origin	Hepatomegaly or right upper quadrant painof liver origin	Hepatomegaly
	Ascites +/− Unexplained weight gain	Unexplained weight gain >2% baseline due to fluid accumulation in the body	Ascites
			Weight gain ≥5% baseline

D = day, HCT = hematopoietic stem cell transplantation.

**Table 3:** McDonald severity grading classification for HVOD.

Severity Grading Classification of HVOD	Criteria
Mild disease	No apparent adverse effect from liver disease. No medications for diuresis of excessive fluid or for hepatic pain. Completely reversible signs, symptoms, and laboratory abnormalities.
Moderate disease (≥1 criteria)	Adverse effect from liver disease. Need to sodium restriction and diuretics to minimize signs of fluid excess (edema, ascites, cardiopulmonary congestion) or medication to alleviate pain from hepatomegaly. Complete resolution of all signs of liver damage (a return of weight to baseline, a decrease in liver size, and a decrease in total serum bilirubin to <34.2 µmol/L (2 mg/dL).
Severe disease (2 criteria)	Adverse effect from liver disease. No resolution of signs, symptoms and laboratory values before D+100. Death.

HVOD = hepatic veno-occlusive disease, D = days.

**Table 4:** Grade of severity of HVOD in children, adolescents, and young adults according to 2019 EBMT recommendations.

Severity Grading of the Disease	Mild (Grade I)	Moderate (Grade II)	Severe (Grade III)	Very Severe (Grade IV)	Death
CTCAE	1	2	3	4	5
Liver enzymes (AST, ALT, GLDH)	≤2× normal	>2 a ≤5× normal		>5× normal	
Bilirubin (mg/dL)	<2		≥2		
Bilirubin (µmol/L)	<34		≥34		
Coagulopathy (not responsive to vitamin K administration, INR value )	<1.5	1.5–1.9	>2	Need for replacement of coagulation factors	
Ascites	Mild (minimal fluid by liver, spleen, or pelvis)	Moderate (<1 cm fluid)	Severe (fluid in all three regions, with fluid collection >1 cm in at least 2 regions)	Need for paracentesis (external derivation of ascites)	
Weight gain (from baseline)	2–5%	5–10% despite diuretic use	>10%	Persistent rise	
Renal function score	KDIGO 1: serum creatinine 1.5–1.9 × baseline or ≥26.5 mmol/L (≥0.3 mg/dL) increase or urine output <0.5 mL/kg/h for 6–12 h	KDIGO 2: serum creatinine 2.0–2.9 × baseline or urine output <0.5 mL/kg/h for ≥12 h	KDIGO 3: serum creatinine 3.0 × baseline or increase in serum creatinine ≥353.6 mmol/L (≥4.0 mg/dL) or initiation of renal replacement therapy or decrease in eGFR to <35 mL/min per 1.73 m^2^ (patients <18 years) or urine output <0.3 mL/kg/h for ≥24 h or anuria for ≥12 h (patients <18 years)	Need for renal replacement therapy	
Encephalopathy	CAPD <9		CAPD ≥9		
Persistent RT	<3 days	3–7 days		>7 days	
Pulmonary function (need for oxygen therapy)	<2 L	>2 L	Non-invasive/invasive mechanical ventilation	Invasive mechanical ventilation	

ALT = alanine transaminase, AST = aspartate transaminase, CAPD = Cornell assessment of pediatric delirium, CTCAE = common terminology criteria for adverse events, eGFR = estimated glomerular filtration rate, GLDH = γ-glutamyl transferase, INR = international normalized ratio, KDIGO = Kidney disease: improving global outcomes, RT = refractory thrombocytopenia.

**Table 5:** Characteristics of study cohort.

	2014–2017	2018–2021
Number of patients	82	97
Number of HCT	89	108
Number of HVOD patients	10	16
Gender of HVOD patients		
Male	3 (30%)	6 (37.5%)
Female	7 (70%)	10 (62.5%)
Age of HVOD patients (years)	1.2–19 (median: 10.3)	1.6–16 (median: 5)
Diagnosis of HVOD patients		
B-ALL	4 (40%)	1 (6.2%)
T-ALL	0	1 (6.2%)
MDS	1 (10%)	2 (12.5%)
AML	0	2 (12.5%)
JMML	1 (10%)	1 (6.2%)
MBL	0	1 (6.2%)
NBL	1 (10%)	8 (50%)
WAS	1 (10%)	0
CAMT	1 (10%)	0
VSAA	1 (10%)	0
Type of HCT in HVOD patients		
autologous	1 (10%)	9 (56.2%)
allogeneic	9 (90%)	7 (43.8%)
MUD	8	7
MSD	1	0
Source of HSC in HVOD patients		
PBSC	6 (60%)	12 (75%)
BM	3 (30%)	4 (25%)
BM + UCB	1 (10%)	0
Conditioning regimen in HVOD patients		
MAC	7 (70%)	14 (87.5%)
RIC	3 (30%)	2 (12.5%)

ALL = acute lymphoblastic leukemia, AML = acute myeloid leukemia, BM = bone marrow, CAMT = congenital amegakaryocytic thrombocytopenia, HSC = hematopoietic stem cells, HCT = hematopoietic stem cell transplantation, JMML = juvenile myelomonocytic leukemia, MAC = myeloablative conditioning regimen, MBL = medulloblastoma, MDS = myelodysplastic syndrome, MSD = matched sibling donor, MUD = matched unrelated donor, NBL = neuroblastoma, PBSC = peripheral blood stem cell, RIC = reduced-intensity conditioning, UCB = umbilical cord blood, VSAA = very severe aplastic anemia, WAS = Wiskott-Aldrich syndrome.

**Table 6:** Statistical comparison of HVOD study cohorts.

Monitored Parameters	2014–2017	2018–2021	Statistical Comparison
Incidence of HVOD	10 (11.2%)	16 (14.8%)	Chi-square = 0.55 *p* = 0.46
Symptoms and signs at diagnosis			
RT	9 (90%)	13 (81.2%)	
Weight gain	8 (80%)	7 (43.7%)	
Hepatomegaly	10 (100%)	12 (75%)	
Ascites	8 (80%)	14 (87.5%)	
Elevation of serum bilirubin level (>34 µmol/L = 2 mg/dL or increase of serum bilirubin level from baseline during first three days following)	6 (60%)	9 (56.2%)	
US signs of HVOD at diagnosis			
Classical US	10 (100%)	15 (93.7%)	
Doppler US	5 (50%)	13 (81.2%)	
Time of HVOD diagnosis post-HCT	6–35 days (median: 15.6 days)	2–23 days (median: 15.7 days)	U = 74 *p* = 0.75
Early HVOD	9 (90%)	13 (81.2%)	
Late-onset HVOD (according to D+21 after HCT)	1 (10%)	3 (18.7%)	
Anicteric HVOD at diagnosis	5 (50%)	14 (87.5%)	Chi-square = 4.40 *p* = 0.04
Serum bilirubin concentration at diagnosis	7.2–234.6 µmol/L = 0.42–13.72 mg/dL (median: 58.2 µmol/L = 3.4 mg/dL)	2.9–87.4 µmol/L = 0.17–5.11 mg/dL (median: 21 µmol/L = 1.23 mg/dL)	U = 42 *p* = 0.045
Maximum serum bilirubin concentration during the HVOD course	7.2–544.1 µmol/L = 0.42–31.82 mg/dL (median: 163.9 µmol/L = 9.58 mg/dL)	17.7–520 µmol/L = 1.04–30.41 mg/dL (median: 73.3 µmol/L = 4.29 mg/dL)	U = 53 *p* = 0.17
Severity grade of HVOD			
I	2 (20%)	5 (31.2%)	
II	5 (50%)	4 (25%)	
III	3 (30%)	1 (6.2%)	
IV	-	6 (37.5%)	
MOD/MOF			
Coagulopathy	5 (50%)	4 (25%)	
Oxygen inhalation therapy	5 (50%)	2 (12.5%)	
Ventilation support	3 (30%)	1 (6.2%)	
Renal insufficiency	2 (20%)	1 (6.2%)	
Encephalopathy	3 (30%)	2 (12.5%)	
Duration of defibrotid treatment	5–43 days (median: 21.7 days)	7–30 days (median: 15.6 days)	U = 73.5 *p* = 0.73
CR of HVOD with defibrotid treatment	7 (70%)	15 (93.7%)	
OS D+100	7 (70%)	15 (93.7%)	Chi-square = 2.67 *p* = 0.10
Mortality D+100	3 (30%)	1 (6.2%)	Chi-square = 2.67 *p* = 0.10
Length of hospitalization	30–134 days (median: 73.1 days)	39–88 days (median: 59.6 days)	U = 68.5 *p* = 0.54

CR = complete resolution, D = day, HCT = hematopoietic stem cell transplantation, HVOD = hepatic veno-occlusive disease, MOD/MOF = multi-organ dysfunction/multi-organ failure, OS = overall survival, RT = refractory thrombocytopenia, US = ultrasound.

## Data Availability

No publicly available datasets are available.
